# Photocurrent Enhancement by Copper Incorporation in Chemical-Solution-Synthesized Inorganic Lead Perovskite Thin Films

**DOI:** 10.1021/acsomega.3c09053

**Published:** 2024-03-22

**Authors:** Igor Borges-Doren, Dagoberto Cabrera-German, Rodrigo Melendrez-Amavizca, Hailin Hu, Mérida Sotelo-Lerma

**Affiliations:** † Departamento de Investigación en Polímeros y Materiales, Universidad de Sonora, Hermosillo 83000, Mexico; ‡ Departamento de Investigación en Física, Universidad de Sonora, Hermosillo 83000, Mexico; § Instituto de Energías Renovables, Universidad Nacional Autónoma de México, Temixco, Morelos 62580, Mexico

## Abstract

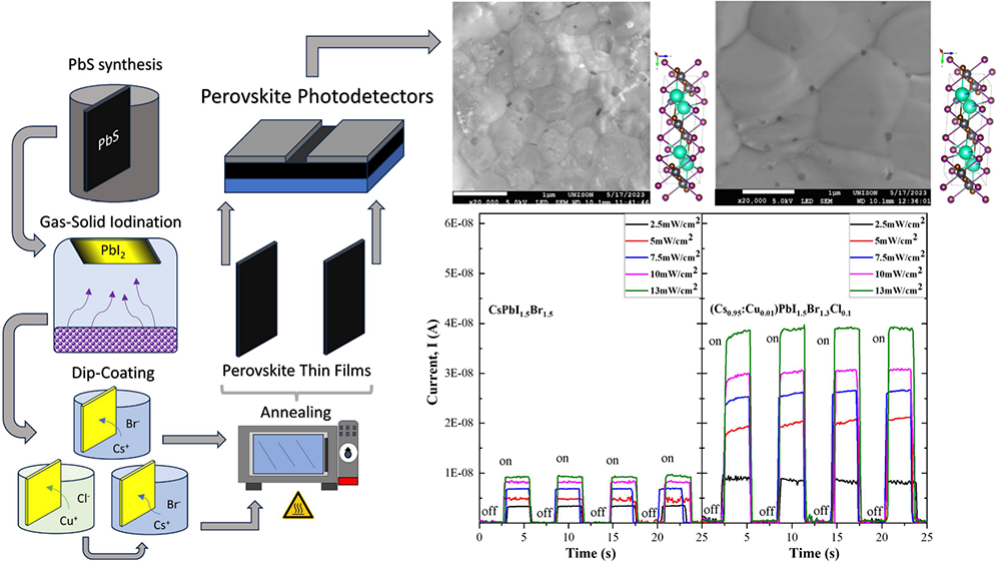

Perovskite thin films are at the forefront of highly promising photovoltaic technologies due to their remarkable optoelectronic properties. Herein, we explore a low-cost, reproducible, and industry-scalable methodology to synthesize an all-inorganic CsPbI_1.5_Br_1.5_ perovskite thin film with additional incorporation of copper and chloride ions into the lattice structure. The synthesis process involves chemical bath deposition of PbS, followed by a gas–solid iodination reaction to yield PbI_2_. Subsequently, dip-coating incorporates Cs^+^, Cu^2+^, Br^–^, and Cl^–^ ions into PbI_2_, and annealing at 270 °C produces perovskite thin films. The results show a large coverage area and a uniform thickness of each perovskite thin film. Comprehensive characterization, including X-ray diffraction, Raman spectroscopy, X-ray photoelectron spectroscopy, scanning electron microscopy, and photoluminescence, provides the structural, chemical, and optical properties of the synthesized thin films. To evaluate the practical implications of our methodology, we fabricated photodetectors employing CsPbI_1.5_Br_1.5_ and (Cs_0.95_:Cu_0.01_)PbI_1.5_Br_1.3_Cl_0.1_ perovskite films. A comparative analysis unequivocally demonstrates a significant increase in photodetector performance when utilizing (Cs_0.95_:Cu_0.01_)PbI_1.5_Br_1.3_Cl_0.1_ perovskite films. While our findings quantitatively assess the tangible enhancement in photocurrent, we acknowledge the potential for improvement in device fabrication to enhance the overall performance. This study not only affirms the successful low-cost synthesis of perovskite thin films but also emphasizes the pivotal role of Cu^2+^ and Cl^–^ ions in enhancing the performance of perovskite-based optoelectronic devices.

## 1 Introduction

Perovskites belong to a family of materials with a characteristic ABX_3_ crystallographic structure, which have gained prominence in various applications due to their unique properties.^1^ In this family, A represents a monovalent cation, B is a divalent metal cation, and X is typically an anion,^2^ with diverse choices for each position. While organic cations like methylammonium CH_3_NH_3_ (MA)^3,4^ and formamidinium CH(NH_2_)_2_ (FA)^5^ have been extensively studied, Cs^+^ ions have emerged as ideal for synthesizing more stable inorganic perovskites.^6^ The crystalline structure of perovskites must satisfy the Goldschmidt tolerance factor (t) and the octahedral factor (μ)^7^ conditions, both fulfilled by Cs^+^, Pb^2+^, and a combination of Br^–^ and I^–^.^8^

In perovskite thin film synthesis, the controlled inclusion of copper ions has shown promise in enhancing grain sizes and increasing free carriers,^9,10^ thereby improving the efficiency of the devices, particularly solar cells.^11^ However, the properties of the perovskites are greatly influenced by the synthesis method, according to numerous reports^12−16^, and the synthesis process proposed here aims to leverage the unique advantages of our three-step methodology, that is control over the chemical composition, reproducibility, use of nontoxic solvents, and a simple, cost-effective technique. Our motivation stems from the increasing use of perovskite films in photodetector devices,^17^ particularly in photodiodes,^18^ phototransistors,^19^ and photoconductors.^20^

The significance of this work extends beyond conventional film synthesis; our approach addresses critical concerns associated with the use of toxic solvents and the fabrication process. Unlike methods employing hazardous solvents such as DMF/DMSO,^21,22^ our low-cost solution-based methodology minimizes the environmental impact and rapid crystallization processes.^23^ Despite being more complex than one-step routes, which often involve toxic solvents or surfactants, the reduction of potential hazards in our three-step approach is a crucial consideration. Moreover, our proposed synthesis method offers a distinct advantage in facile utilization during photolithography patterning steps. Notably, our synthesis methodology might exhibit resilience during direct etching, as our precursor films, PbS and PbI_2_, can withstand the etching steps required in device fabrication,^24^ impacting the stability and structural integrity of perovskite materials.

Thus, we present a methodology for synthesizing all-inorganic perovskite materials, showcasing noteworthy optoelectronic properties in photodetector devices. Our three-step methodology involves a final dip-coating step to incorporate Cs^+^ cations, Br^–^, and Cl^–^ anions into a PbI_2_ film, varying the stoichiometry of copper and chloride ions in the films. To control the copper amount, we varied the concentration of CuCl salt in different solutions, immersing the films obtained in the previous stage in each of these solutions. Importantly, we observed a positive effect on increasing the photocurrent with the incorporation of copper and chloride ions.

## 2 Experimental Details

### 2.1 Materials

First, the proposed methodology follows the initial synthesis of a PbI_2_ thin film consisting of a two-step process as successfully reported elsewhere.^25,26^ The reagents employed to chemically synthesize PbS thin films were lead(II) acetate trihydrate, Pb(CH_3_COO)_2_·3H_2_O (FAGALAB, assay 99.82%); thiourea, (NH_2_)_2_CS (Fermont, assay 99.1%); triethanolamine (TEA), N(CH_2_CH_2_CH_2_OH)_3_ (FAGALAB, 99.6% purity); distilled water, and NaOH (Fermont, assay 97.8%). In the iodination reaction, solid iodine (FAGALAB, 99.9% purity) was used. The incorporation of the metallic and halide ions was done in a dip-coating solution using CsBr (Aldrich, assay 99.9%) and CuCl (Meyer, assay >90.0%).

The PbS thin film was deposited onto a glass substrate VIAND Cat. 7102 microscope glass slides (0.8–1.1 × 26 × 76 mm). These glass substrates were cleaned before deposition, consisting of a thorough wash with Alconox detergent and a subsequent rinse with distilled water until no apparent traces of detergent were perceived. Only one side of the glass slide was exposed to the chemical reaction by covering the other side with adhesive tape.

### 2.2 Perovskite Synthesis Methodology

**Figure 1:**
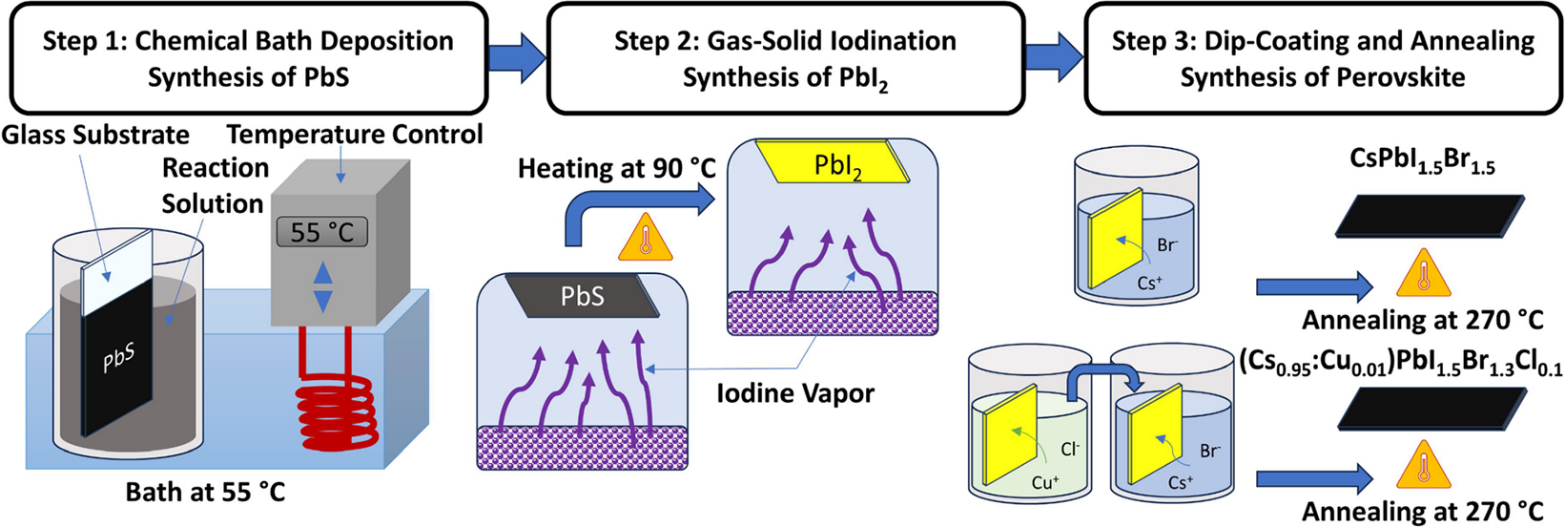
Schematic diagram illustrating the comprehensive synthesis methodology for perovskite films via the PbS-to-PbI_2_-to-XPbI_*x*_Br sequential process.

The inorganic perovskite thin films obtained in this work follow a simple methodology that does not require complex instrumentation. The complete methodology is schematically represented in Figure 1. The first two steps consist of synthesizing a lead sulfide thin film and then iodinating the film via a gas–solid reaction. The reaction solution was prepared following a previous report.^25^ In this case, lead sulfide thin films were prepared by submerging the glass substrates in the reaction solution for 60 min at a constant reaction temperature of 55 °C. The obtained films were washed with distilled water, then cleaned in an ultrasonic bath for 10 min, and subsequently dried at room temperature. This process enabled the deposition of the thin film composed of PbS_0.96_ as determined by X-ray energy dispersive spectroscopy.

The iodination procedure consisted of letting the PbS_0.96_ thin film react with iodine vapor inside an 80 cm^3^ hermetic glass chamber sublimating the iodine at a temperature of 90 °C for 1 h. Afterward, the glass chamber was let to cool at room temperature for 1 h, and then the lead iodide film was removed from the chamber and cleaned with a cool air gun. The film produced has a PbI_1.98_ chemical composition as determined by X-ray energy dispersive spectroscopy.

The XPbI_*x*_Y perovskite thin films were produced by letting cations X (Cs^+^ and Cu^2+^), and halides Y (Br^–^ and Cl^–^) incorporate into the PbI_1.98_ film via dip-coating technique and subsequently heating the films at 270 °C in a muffle having an air atmosphere for 30 min and letting them cool afterward until room temperature is reached for 3 h. The purpose of the annealing is to eliminate the solvent excess and guarantee the most crystalline possible formation of the perovskite films. The CsPbI_1.5_Br_1.5_ perovskite thin film was synthesized by submerging the PbI_1.98_ film in a reaction solution composed of 0.25 M CsBr. The perovskite that incorporates Cu^2+^ and Cl^–^ in the structure was done by submerging first the PbI_1.98_ film into an isopropyl alcohol solution having CuCl concentrations of 2.5 mM up to 10.0 mM and then dip-coating the film in the 0.25 M CsBr solution. The dip-coating was done for 10 s in each step.

### 2.3 Photodetector Device Fabrication

The photocurrent response performance of an inorganic perovskite thin film was evaluated in a photodetector device, which was fabricated as follows: the perovskite thin films were cut from the original glass slide into equal square pieces having a 2.00 cm^2^. Then a pair of silver-paint contacts (DuPont colloidal silver paint) with an area of 0.98 cm^2^ and a separation of 0.2 mm were painted on top of the perovskite film, permitting an effective illumination area of 2.0 mm^2^. The assessment of the perovskite photodetector involved the use of a tungsten-halogen lamp at room temperature as a source of illumination with five different light intensities going from 2.5 up to 13 mW/m^2^. A Keithley 2400 device was employed to record the current while applying a voltage of 5.0 V. This process involved recording current values for 2.5 s in the absence of light (referred to as dark current), followed by 5 s of exposure to illumination (referred to as light current), and then another 5 s of dark current measurement. This switching cycle was repeated four times.

### 2.4 Material Characterization

The surface characteristics of the perovskite thin films were assessed via field emission scanning electron microscopy (FESEM) with a JEOL JSM-7800F microscope recording backscattered electrons. An X-ray energy dispersive analyzer coupled to the FESEM tool provided energy dispersive spectroscopy data. X-ray diffraction (XRD) data was recorded using a Rigaku D8Advance eco diffractometer equipped with a Cu Kα = 1.54 Å X-ray source, coupled with a Ni filter operated in a Bragg–Brentano configuration. Raman spectroscopy measurements were obtained using a confocal Raman microscope model Alpha300RA (WITec, Germany) with laser excitation of 532 nm, 10 mW of laser power, and approximately a 1 μm spot size. X-ray photoelectron spectra (XPS) were obtained using a PerkinElmer (PHI division) 5100 spectrometer with a nonmonochromatic Mg Kα X-ray source and recording experimental data with a pass energy of 18 eV; peak-fitting analysis was done using the AAnalyzer software.^27^ Photoluminescence (PL) spectra were obtained via a modular spectrofluorometer, HORIBA Fluorolog model 322, with an excitation laser of 488 nm (Coherent Brand, Obis model), operated at 20 mW.

## 3 Results and Discussion

### 3.1 Surface Morphology of the Perovskite Thin Films

**Figure 2:**
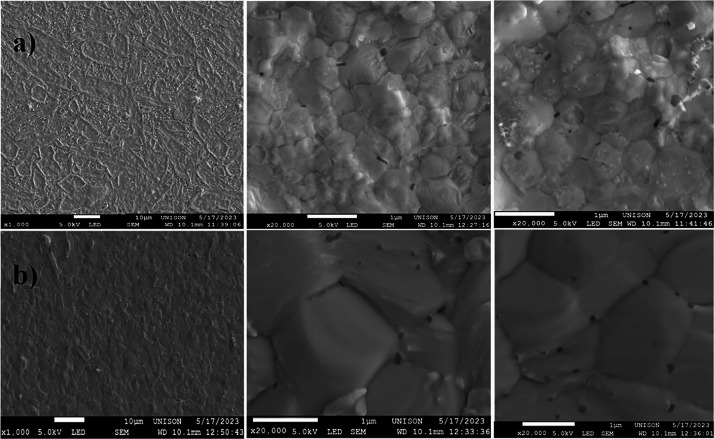
FESEM micrographs of the perovskite thin films with different amplifications (a) CsPbI_1.5_Br_1.5_ thin film and (b) (Cs_0.95_:Cu_0.01_)PbI_1.5_Br_1.3_Cl_0.1_ perovskite. The white bar in each image indicates the scale.

The surface morphology of two perovskite thin films was assessed: one corresponding to the inorganic perovskite film synthesized with only Cs and Br ions in its structure (Figure 2a) and the other incorporating Cu and Cl ions, corresponding to the 2.5 mM dip-coating solution (Figure 2b). The estimated chemical composition via energy-dispersive spectroscopy (EDS) for the first perovskite film is CsPbI_1.5_Br_1.5_ and for the second one, (Cs_0.95_:Cu_0.01_)PbI_1.5_Br_1.3_Cl_0.1_. From Figure 2, we observe that for both perovskite thin films, there is complete coverage of the glass substrate with no delamination of the films for the 1kx amplification, showing that the present methodology is viable for large-area coatings. In further amplification, we observe that both CsPbI_1.5_Br_1.5_ and (Cs_0.95_:Cu_0.01_)PbI_1.5_Br_1.3_Cl_0.1_ perovskite thin films have a well defined microstructure characterized by large, compact, and prismatic grains, as reported elsewhere.^15,23^ The grain sizes were estimated with ImageJ, resulting in compact grains of around 0.49 μm in the case of the CsPbI_1.5_Br_1.5_ thin films. While the incorporation of copper and chloride ions seems to enable an increase in the compact grain size up to 1.29 μm, a phenomenon that matches with reported literature,^28,29^ a major contribution to the improvement of the photoconductivity response is observed due to fewer grain boundaries. It is important to note that pores are present as we get a closer look at the surface of the films; however, they are not pinholes that reach down to the glass substrate. This microporous morphology may be evidence suggesting that in sections of the film, some solvents accumulated at the surface of the film, vaporize during annealing until they burst out into the atmosphere.

**Figure 3:**
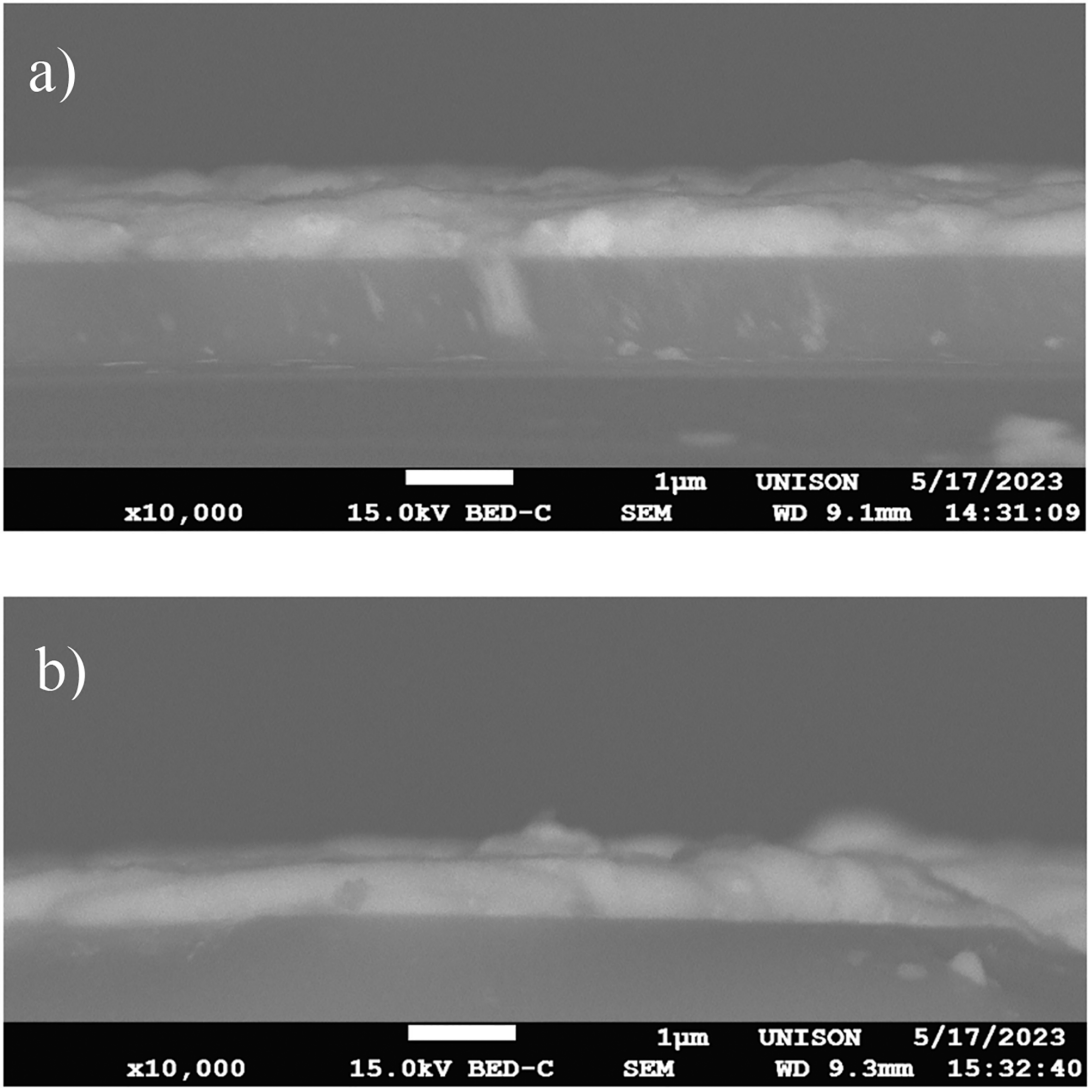
FESEM cross-section of the (a) CsPbI_1.5_Br_1.5_ and (b) (Cs_0.95_:Cu_0.01_)PbI_1.5_Br_1.3_Cl_0.1_ perovskite thin films. The white bar in each image is 1 μm.

Figure 3 shows the FESEM cross-sectional images of the perovskite thin films. The average thickness of the CsPbI_1.5_Br_1.5_ thin film lies around 554 nm, while for the (Cs_0.95_:Cu_0.01_)PbI_1.5_Br_1.3_Cl_0.1_ perovskite, the thickness increases to 570 nm with the incorporation of CuCl.

### 3.2 Structural Characteristics

**Figure 4:**
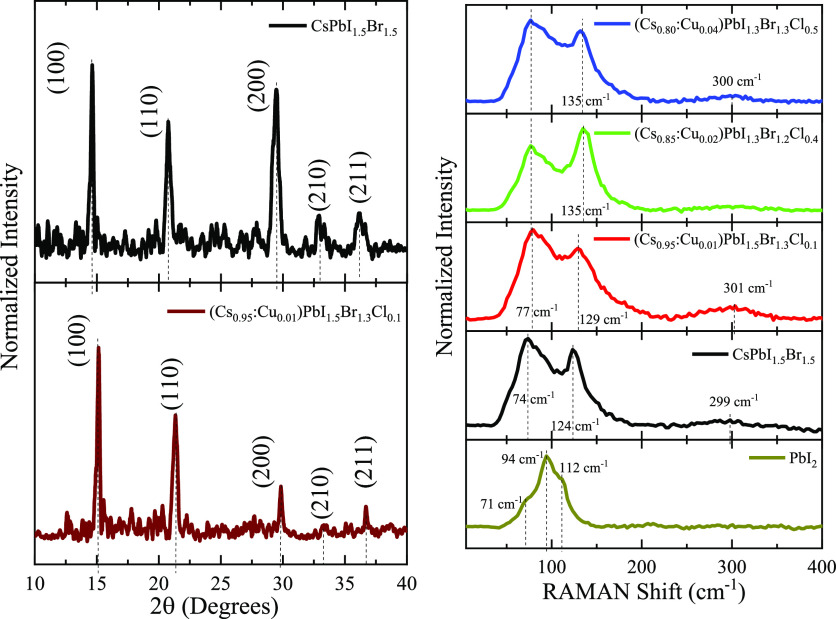
Left shows the X-ray diffraction patterns of the CsPbI_1.5_Br_1.5_ and (Cs_0.95_:Cu_0.01_)PbI_1.5_Br_1.3_Cl_0.1_ perovskite thin films. The right section shows the RAMAN spectra of the all-inorganic perovskite thin films obtained at varying cation and halide incorporation, along with the RAMAN spectrum of the PbI_2_ precursor film as a reference.

The structural characteristics of the perovskite thin films were assessed by utilizing XRD and Raman spectroscopy. The XRD data is presented in the left section of Figure 4 where five diffraction peaks are observed. The diffraction peaks observed for both perovskite thin films are indexed to the (100), (110), (200), (210), and (211) planes of a cubic perovskite structure, with a *Pm-3m* space group, as previously reported in the literature.^30−32,13,33^

For the CsPbI_1.5_Br_1.5_ thin film, the relative intensities of the diffraction peaks diverge from those of the pure CsPbI_2_Br, CsPbBr_3_, or CsPbI_3_ phases. Instead, the observed peak positions and relative intensities approximate the weighted average of the cubic phases CsPbBr_3_ and CsPbI_3_.^31^ Considering the assessed chemical composition, it is plausible to propose that the observed diffraction pattern is indicative of a cubic CsPb(Br_*x*_I_1–*x*_)_3_ perovskite solid solution with *x* = 0.5.

It is interesting to observe that the perovskite film that incorporates Cu and Cl into the structure has its three main diffraction peaks shifted 0.5 ± 0.1 degrees to higher angles of 2θ, evidence that might suggest that the (Cs_0.95_:Cu_0.01_)PbI_1.5_Br_1.3_Cl_0.1_ perovskite has induced stress that compresses the unit cell caused by the different ionic radii of Cu, Cs, and Pb.^10,11^ Moreover, the relative intensities of the diffraction peaks do not correspond to the weighted average of the cubic phases CsPbBr_3_ and CsPbI_3_; instead, we observe that the (100) peak is exacerbated, and the (200) peak has a reduced intensity. This is a texturing effect that perhaps is the result of the Cu doping, where Cu incorporates interstitially into the (100) crystal facets, restricting the crystal growth over the <100> direction, which also leads to a compression of the unit cell as evidenced by the diffraction peak shift. The preferential growth of perovskites has been reported before,^30,31^ where the observed contraction of the unit cell and the altered peak intensity of the (100) peaks, highlight the intricate interplay between the Cu doping, crystal facet interactions, and the resultant texturing effects, perhaps even in some regions of the film some copper ions substitute the Pb,^34,35^ leading to the reduced intensity of the (200).

As observed in the FESEM results, the (Cs_0.95_:Cu_0.01_)PbI_1.5_Br_1.3_Cl_0.1_ perovskite thin film exhibits larger grain sizes, which can be corroborated by estimating the crystallite size using the Scherrer method^36^ for the (100) plane. The average crystallite size is 23 nm, while for the CsPbI_1.5_Br_1.5_ thin film, the crystallite is approximately 19 nm. These results comply with existing literature,^7^ where the incorporation of copper ions into the perovskite structure is linked to an increase in crystallite size. In this case, the introduction of copper ions is proposed to decrease the crystallization rate,^10^ allowing the growth of large grains instead of the rapid formation of crystallites that typically yield smaller grains in comparison. Also, the introduction of copper ions fundamentally alters the crystalline structure due to the difference in atomic radii between copper, cesium, and lead that induces stress and preferential growth in the perovskite material.

The Raman spectra for the perovskite thin films are found in the right section of Figure 4, with the PbI_2_ spectrum included as a reference, displaying the signature peaks of the 2H–PbI_2_ type structure.^37,38^ In the case of the CsPbI_1.5_Br_1.5_ thin film, the spectrum shows a sharp Raman band at 124 cm^–1^ and two broad bands at 74 and 299 cm^–1^, which are related to the transverse-optical (TO) and longitudinal-optical (LO) modes of the cubic perovskites.^39−42^ The peak at 74 cm^–1^ is related to the TO_2_ mode, the narrow band at 124 cm^–1^ corresponds to the TO_3_ mode, and the broad band at 299 cm^–1^ corresponds to the second-order phonon LO_2_ mode. Although the LO_1_ mode is not observed as a distinct peak due to the strong overlap with the TO modes, a broad shoulder around 150 cm^–1^ can be attributed to this phonon mode.

We can observe that as Cu and Cl are incorporated into the perovskite thin films, the Raman bands shift to higher wavenumbers, indicating a compressive stress,^41^ consistent with the XRD results. It is noteworthy that the TO_2_ mode associated with the vibration of the [PbX_6_] octahedron, shifts to 77 cm^–1^ and then maintains its position despite the increasing copper and chloride incorporation, while the TO_3_ shifts to 129 cm^–1^ for the (Cs_0.95_:Cu_0.01_)PbI_1.5_Br_1.3_Cl_0.1_ perovskite thin film. With continued doping, the peak reaches 135 cm^–1^, possibly suggesting that the motion of Cs ions^39^ is affected by the copper and chloride incorporation. The results imply that Cu interstitially incorporates into the perovskite affecting the motion of Cs, with a reduced proportion of Cu being able to substitute Pb in the lattice.^34,35^ Also, the broadening can indicate that Cl substitutionally forms Pb–Cl bonds, thereby changing the crystal symmetry of the original perovskite.

### 3.3 Chemical State Assessment

**Figure 5:**
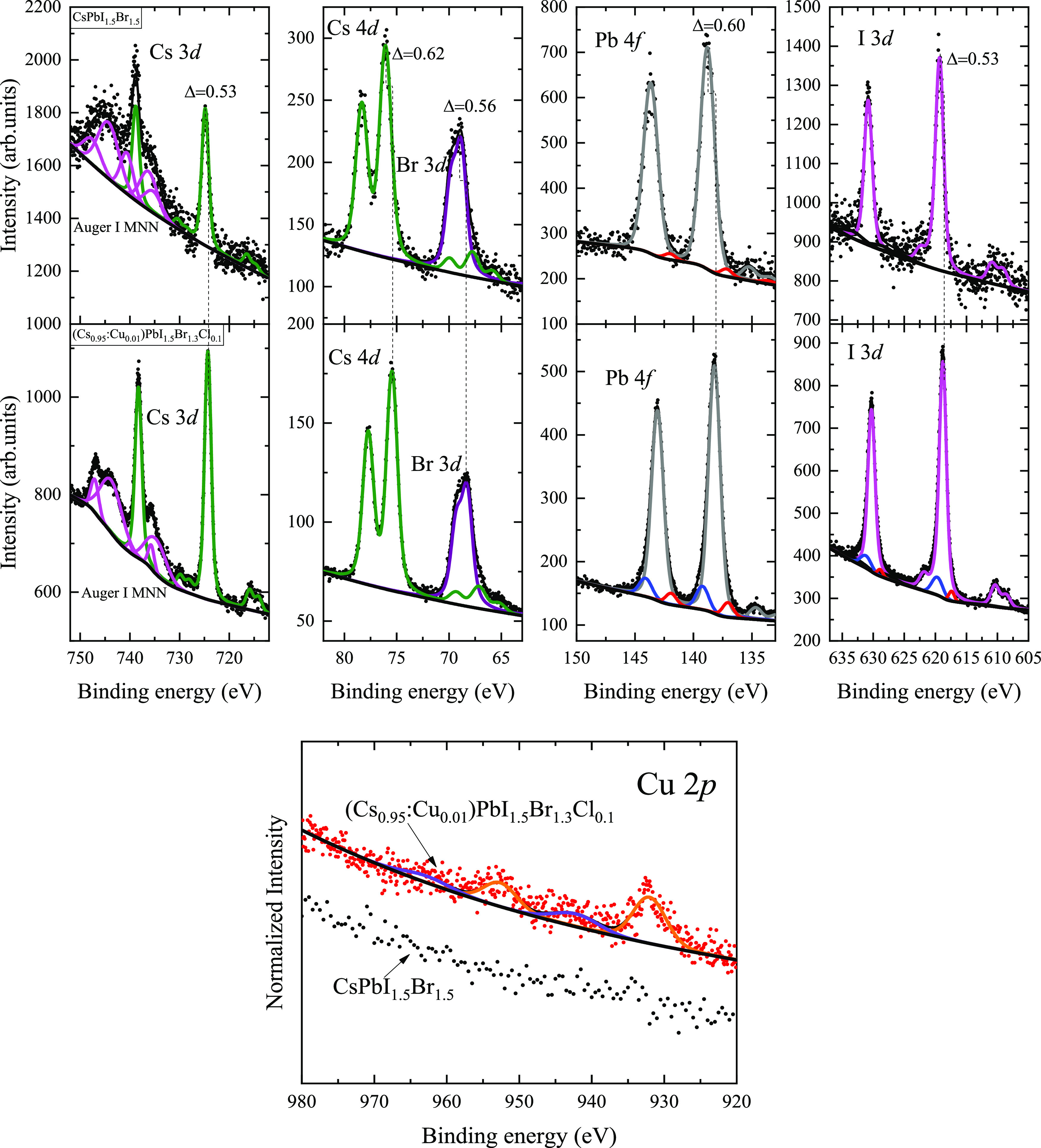
X-ray photoelectron spectra of CsPbI_1.5_Br_1.5_ and (Cs_0.95_:Cu_0.01_)PbI_1.5_Br_1.3_Cl_0.1_ perovskite thin films. Each spectrum corresponds to the principal atomic components of the films.

The chemical characteristics of both perovskite thin films are presented in Figure 5. Here, we can observe the principal photoelectron signals associated with the presence of the atoms composing each of the perovskite thin films. For the CsPbI_1.5_Br_1.5_ perovskite, the Cs 3d_5/2_ and I 3d_5/2_ signals are energy located at 724.80 and 619.29 eV binding energy, respectively, which are in correspondence to other reports in the literature.^10^ Also, we can observe the presence of the Pb 4f_7/2_ located at 138.79 eV, associated with a Pb^2+^ state^43^ and a low-intensity signal related to metallic Pb.^38^ The binding energy values of the Br 3d_5/2_ and Cs 4d_5/2_ are found to be 68.81 and 76.05 eV, which are similar results to what various authors have reported elsewhere.^44^

By analyzing the photoelectron spectra for the (Cs_0.95_:Cu_0.01_)PbI_1.5_Br_1.3_Cl_0.1_ perovskite thin film, there exists evidence supporting that Cu atoms induced a change in the local chemical environment of the perovskite, which consists in an overall chemical shift to lower binding energies of around 0.57 ± 0.08 eV for all photoelectron signals. This shift to lower binding energies has also been observed in other works where Cu atoms are incorporated into a perovskite structure.^10^ The individual chemical shifts for each photoemission line are listed in Figure 5.

It is important to note that there are also subtle changes in the low-intensity signals in all of the spectra. In the case of the Cs 3*d* core level, the Auger I MNN complex structure changes completely in the perovskite thin film having copper and chloride, suggesting that the electronic structure is affected by the copper and chloride incorporation. In correspondence, the I 3d spectrum also shows two new peaks, on both the higher and the lower binding energy side of the main photoemission peak, suggesting that there may be iodides within the perovskite unit cell that have less electronic density in the case of the higher binding energy side peak and other iodides with more electronic density in the case of the lower binding energy side peak, meaning that local chemical environment of the iodides are more clearly affected by the presence of Cu and Cl in this perovskite. The Pb 4f also shows the presence of two additional signals in the case of the (Cs_0.95_:Cu_0.01_)PbI_1.5_Br_1.3_Cl_0.1_ perovskite thin film, although in this case, the peaks have been reported before as coming from the formation of a minoritarian lead oxide^25,38^ at the surface of the film and the remanent presence of metallic lead.^38^

Further corroboration for copper incorporation into a perovskite structure is done by analyzing the Cu 2p spectra (the Cl 2p spectra are not shown due to the relatively low photoionization cross-section^45^ and concentration of this atom, not allowing the measurement of quality experimental data), where for the CsPbI_1.5_Br_1.5_ film, no Cu 2p photoemission peaks are detected. In contrast, for the (Cs_0.95_:Cu_0.01_)PbI_1.5_Br_1.3_Cl_0.1_ perovskite thin film, there is a clear presence of a main photoelectron peak located at 931.99 eV binding energy and a strong satellite feature at 942.19 eV binding energy; these strongly suggest that the chemical state of copper in the perovskite is a Cu^2+^ state.^46,47^ This result is very interesting because it suggests that the Cu^+^ ions are oxidized to Cu^2+^ as it is incorporated into the perovskite structure, a result that might support the idea that the incorporation of copper into the perovskite structure follows an interstitial doping or substitutional incorporation between the lead ions.^34,35^

A reason for this oxidation might be that a thermally stable perovskite structure is formed with the inclusion of Cu^2+^ ions^48^ where the incorporation of Cu^2+^ is facilitated by the structural compatibility and chemical bonding between the oxidized copper ions and the host material.^48^ However, it is difficult to precisely describe the oxidation of Cu^+^ to Cu^2+^, as it can occur through various mechanisms, where the different solvation structures can also potentially influence the behavior of the copper ions.^49^ In this case, while copper ions are less readily oxidized in nonaqueous solvents compared to aqueous media, it is plausible that oxidation occurs, allowing for the incorporation of Cu^2+^ ions by ionic exchange into the PbI_2_ film during the dip-coating process. This hypothesis is proposed due to the lack of experimental evidence supporting the claim, and the complex nature of the oxidation mechanism, which varies significantly across different thin film systems where both Cu^+^ and Cu^2+^ can coexist.^46,47,50,51^ Subsequently, another possibility is that the Cu^+^-incorporated PbI_2_ film enables the synthesis of the perovskite film by thermal transformation, where the oxidation of copper might also occur.^52^ While direct evidence for the electron transfer leading to the oxidation of copper ions may not be readily observed due to the low copper content in the perovskite, the shift to lower binding energies suggests a slight excess of delocalized electrons in the Cu-incorporated perovskite. These excess electrons may originate from the Cu^+^ ions oxidizing to Cu^2+^, providing electrons to the perovskite, thereby influencing the overall potential felt by other ions in the perovskite structure. Potentially, the perovskite acts as a reducing environment for copper, as evidenced by the observed chemical shifts in the photoelectron spectra, following the previous hypothesis of spontaneous oxidation of Cu^+^ to Cu^2+^ in perovskite materials.^52^ Although Cu^2+^ incorporation is suggested to occur through substitutional incorporation between lead ions to ensure charge compensation of the perovskite structure,^48^ further studies are needed to explore the specific mechanisms and kinetics of this process in perovskite systems. These inquiries extend beyond the scope of our current work, where perhaps a detailed photoemission analysis at various dip-coating solution concentrations and annealing temperatures would be more suitable to address the matter comprehensively. Notably, our identification of the chemical state of copper addresses a gap often overlooked in existing literature reports, which is relevant for understanding the obtained perovskite structure.

### 3.4 Optical and Electrical Characteristics

**Figure 6:**
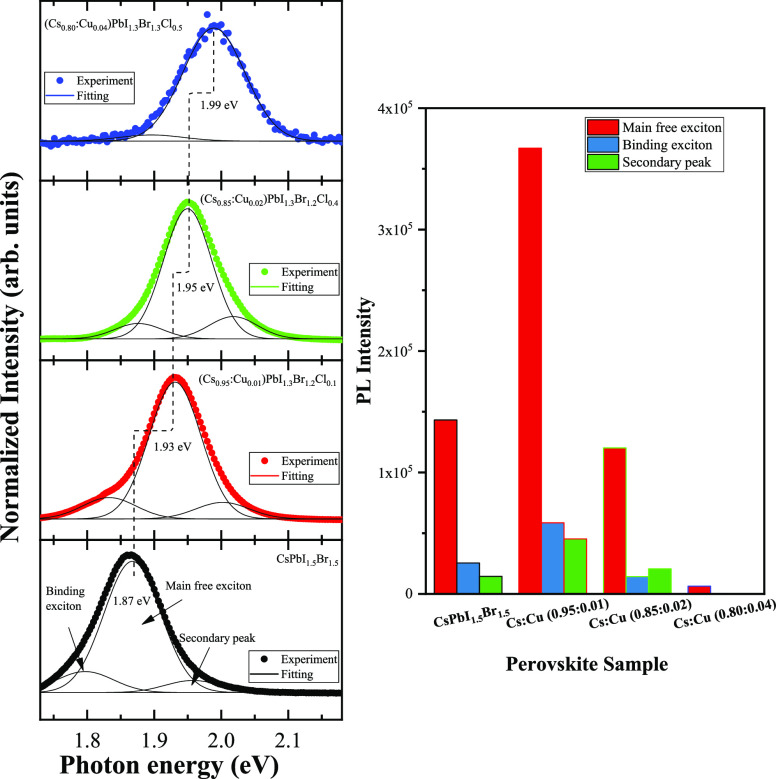
Left shows the normalized PL spectra of perovskite thin films with different copper and chloride content. On the right, the bar graph shows the total integrated intensity that changes according to the Cu content.

The left section of Figure 6 presents the PL spectra for a series of perovskite thin films with varying Cu content. Using the 488 nm laser for excitation, an emission peak centered around 1.87 eV is obtained for the CsPbI_1.5_Br_1.5_ perovskite, consistent with previous reports.^31,53^ As the Cu concentration in the films is increased, the emission maxima shift to higher energies, reaching 1.99 eV. The observed PL blueshift may be related to a possible change in the film stoichiometry as the Cu and Cl content increase and the iodine ions decrease.^54^ On one hand, the incorporation of Cu^2+^ into the perovskite structure may contribute to an increase in free charge carriers, thus shifting the emission maxima.^55^ On the other hand, the blueshift has been attributed to the incorporation of Cl^–^ ions.^56^

Figure 6 shows the peak-fitted PL spectra using Gaussian profiles, where three distinct peaks are required for most samples, the FOM^57,58^ of the fittings for all samples indicate good fits, as shown in Table 1. Particularly, the perovskite sample with the highest Cu incorporation, as determined via EDS, needs only two peaks. The most intense peak may be associated with a PL signal from intrinsic free excitons in the perovskite, whose energy positions align with others reported in the literature for similar perovskites.^59,60^ The peak-fitting also reveals the presence of a lower energy peak related to defect-related bound excitons.^59^

**Table 1:** Curve-Fitting Results of the PL Spectra of the Perovskite Thin Films with Increasing Copper and Chloride Content

sample	peak label	peak position		relative intensity	FOM
		eV	nm	%	%
CsPbI_1.5_Br_1.5_	main free exciton	1.87	664	78	0.93
	secondary peak	1.96	634	8	
	binding exciton	1.80	689	14	
(Cs_0.95_:Cu_0.01_)PbI_1.5_Br_1.3_Cl_0.1_	main free exciton	1.93	642	78	1.05
	secondary peak	2.00	619	10	
	binding exciton	1.83	677	12	
Cs_0.95_:Cu_0.02_)PbI_1.3_Br_1.2_Cl_0.4_	main free exciton	1.95	636	78	1.07
	secondary peak	2.02	614	13	
	binding exciton	1.88	661	9	
Cs_0.95_:Cu_0.04_)PbI_1.3_Br_1.3_Cl_0.5_	main free exciton	1.99	623	95	5.24
	secondary peak				
	binding exciton	1.90	654	5	

Additionally, we detected a PL peak at higher photon energies, labeled as the “secondary peak”. Its nature remains uncertain due to the complexity of the physical processes involved, as most literature PL spectra are fitted with only two peaks. Nonetheless, its presence is significant and cannot be ignored, as it is observed in both Cu-incorporated and Cu-free perovskite films. Under the current experimental conditions, we assume that this emission is associated with a secondary free exciton emission coming from the recombination of photoexcited carriers or emission from the creation of free excitons in compositional-gradient and textured film regions. For example, the PL maxima have been previously reported at 2.39 eV for CsPbBr_3_ and at 1.80 eV for CsPbI_3_ with in-between values for CsPb(Br_*x*_I_1–*x*_)_3_ perovskites with distinct microstructural characteristics,^30^ therefore, the secondary peak might be coming from the radiative recombination of free excitons from regions of the film where there is a prevalence of bromide over the iodide or at grain boundaries. However, a detailed temperature-resolved PL study is deemed necessary for a precise description of these processes.

The PL peaks show a noticeable blue-shift to higher photon energies with increasing Cu and Cl content, primarily attributed to the Cl^–^ incorporation alongside the Cu^2+^ in the perovskite films. This shift may predominantly result from the significantly lower ionic radius of chloride ions compared to the halide iodide ions, probably widening the bandgap due to size differences.^59,61^ Another interpretation involves an increase in the binding energy of the valence orbitals of halide ions, which can also contribute to a widened bandgap.^62^ Despite previous findings^63^ showing that Cu^2+^ incorporation in perovskite films leads to shifts to lower photon energies by sub-bandgap state formation,^63^ in our case, the blueshift effect might be mainly dominated by the inclusion of chloride ions into the perovskite structure.^56^ The increased concentration of Cl^–^ ions compared to Cu^2+^ ions suggests a predominant influence of Cl^–^ ions on the observed blueshift.

Interestingly, the total intensity of the emission spectra is also significantly affected by the incorporation of copper and chloride ions, as can be observed in the right section of Figure 6. First, there is an increase in the total intensity when the atomic Cu content is around 0.01 at %, followed by a drastic decrease as the Cu content increases. The most favorable photoemissive properties are the ones corresponding to the (Cs_0.95_:Cu_0.01_)PbI_1.5_Br_1.3_Cl_0.1_ perovskite thin film, possibly indicating fewer defect-related recombination centers compared to Cu-free perovskite film and films with 0.02 and 0.04 Cu at %.^64,65^ Other reports have shown that the incorporation of chloride is beneficial because it reduces the defects in perovskite materials,^31^ so the observed PL enhancement might be the result of the combined effect of copper and chloride ion incorporation. The reduction in the number of defects might also suggest an increase in the minority carrier lifetimes in copper and chloride-incorporated perovskite, as has been reported in the literature.^59,66^ However, according to the results, an excessive increase in the copper and chloride contents does not lead to the passivation of defects. Instead, it leads to a prevalence of defects, increasing the potential for nonradiative recombination, resulting in a shutdown of the total PL emission.

To determine whether the PL enhancement and subsequent diminution of the spectra are driven by excitonic or defect-related processes, an approach based on spectral contribution ratios was employed.^59,60,67^ The relative intensity of the main free exciton remains around 78% for all samples, except for the Cs_0.85_:Cu_0.04_ perovskite thin film, which yields 95%. In the case of binding exciton emission, the relative intensity monotonically decreases from 14% for the CsPbI_1.5_Br_1.5_ perovskite, 12% for the Cs_0.95_:Cu_0.01_, 9% for the Cs_0.90_:Cu_0.02_, and finally reaching 5% for the Cs_0.85_:Cu_0.04_ perovskite thin film. This trend suggests that the relative intensity of defect-related bound excitons decreases with increasing copper concentration. Conversely, the secondary emission increases (8% for the CsPbI_1.5_Br_1.5_, 10% for the Cs_0.95_:Cu_0.01_, and 13% for the Cs_0.90_:Cu_0.02_ perovskite thin film), suggesting its involvement in unbound electron–hole pairs that recombine via augmented recombination pathways as the electronic structure of the perovskites is modified with the copper and chloride incorporation.

**Figure 7:**
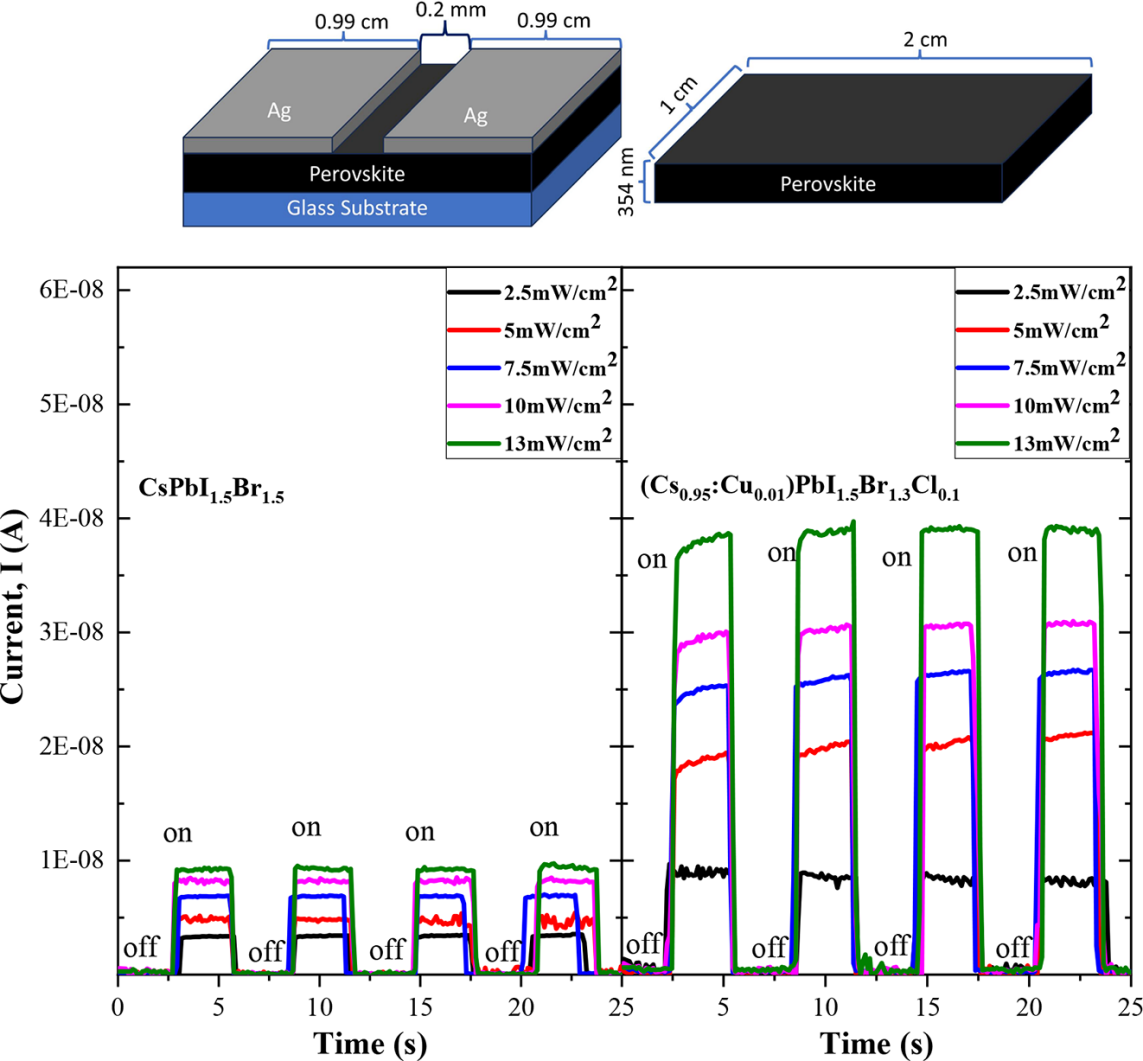
Top section shows the schematic of the photodetector device with an illumination area of 2.0 mm^2^. Conductivity photoresponse of the CsPbI_1.5_Br_1.5_ (Bottom-left) and (Cs_0.95_:Cu_0.01_)PbI_1.5_Br_1.3_Cl_0.1_ (Bottom-right) perovskite thin films with different incident light intensity.

Photoconductivity measurements were conducted to evaluate the photocurrent generated by perovskite-based photodetectors. The schematic of the device design is presented in the top section of Figure 7. Measurements were recorded by varying the light source intensity from 0 to 13 mW/cm^2^ at a constant voltage of 5.0 V. Both sets of fabricated photodetector devices, one using the CsPbI_1.5_Br_1.5_ film and another utilizing (Cs_0.95_:Cu_0.01_)PbI_1.5_Br_1.3_Cl_0.1_, showed a clear response of the current to the incident light, as seen in Figure 7.

The strong influence of the copper and chloride incorporation on perovskite-based photodetectors is evident, with the induced photocurrent being approximately 4 to 5 times larger than that obtained for the CsPbI_1.5_Br_1.5_-based device under the same illumination conditions. Previous reports have noted that doping perovskite materials with divalent ions induces lattice contraction^68^ (in agreement with our XRD and Raman results), significantly reducing the number of defect states.^65^ Additionally, the PL results might suggest that the chloride incorporation^31^ contributes to improving the photocurrent response by surface passivation of surface defects.

The response time for both devices was assessed by comparing the device performance at a 13 mW/cm^2^ light intensity using 1 s time pulses. The response times were found to be adequate, with the CsPbI_1.5_Br_1.5_ film showing a rise time of 39 ms and a fall time of 17 ms. Likewise, for the (Cs_0.95_:Cu_0.01_)PbI_1.5_Br_1.3_Cl_0.1_ perovskite thin films, the rise and fall time results were determined at 38 and 36 ms, respectively. Although these values may not surpass the best reported in the literature,^69^ considering the complexity of their fabrication processes,^70^ our low-cost method offers competitive results, presenting an attractive alternative for device fabrication. Notably, our results compare favorably with other perovskite devices fabricated via low-cost processes^71^ and show slight improvement compared to those using similar methodologies to ours.^25^

**Figure 8:**
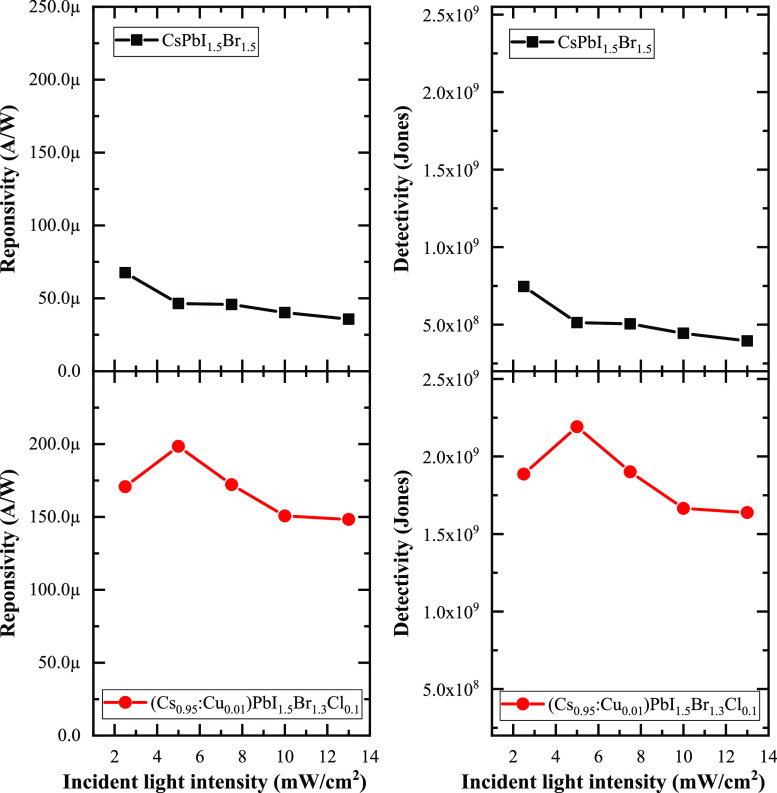
Left presents the responsivity for the CsPbI_1.5_Br_1.5_ and (Cs_0.95_:Cu_0.01_)PbI_1.5_Br_1.3_Cl_0.1_ perovskite thin films with increasing light illumination intensity, while the right section shows the detectivity of the photodetector devices.

The responsivity of the devices is presented in Figure 8, with the effective illuminated area denoted as *A*_L_ = 0.02 cm^2^, used to determine the responsivity as the quotient between the photocurrent, effective area, and illumination intensity.^72^ The results indicate that there is a slight overall decrease in the responsivity from 67 μA/W at 2.5 mW/cm^2^ to 45 μA/W at 13 mW/cm^2^ for the CsPbI_1.5_Br_1.5_ thin film. In contrast, the copper and chloride incorporation in the perovskite film enables the fabrication of a device with responsivity ranging from 160 μA/W at 2.5 mW/cm^2^ to 148 μA/W at 13 mW/cm^2^. These responsivity values prove competitive when compared to other perovskite-based photodetectors fabricated using low-cost solution methods.^25,71^

The detectivity of the devices is determined following a standard procedure,^73,69^ where this figure-of-merit value strongly depends on the device dimension and synthesis method, with composition, morphology, and stability influencing this property, as indicated in numerous reports^74−78^. While our devices exhibit low detectivity values compared to the best in the literature, it is important to note that other perovskite studies often involve elevated temperatures, complex purification steps, or the frequent use of toxic solvents.^79^ The performance of our devices is also notably influenced by contact quality, as in our case, contacts were painted on top. The quality of contacts can lead to carrier loss due to inadequate contact or imprecise patterning. Furthermore, as the devices were not fabricated in a clean-room, surface contamination contributes to suboptimal performance. Despite these challenges, we believe that the perovskite materials presented in this work hold promising potential for device fabrication using our low-cost, solution-based method. While our proposed methodology involves more steps, it eliminates the use of toxic solvents, stabilizing agents, or high temperatures that are incompatible with flexible electronics. In comparison with detectivity values under the same conditions in other reports,^25,71^ our results demonstrate an improvement.

Overall, the results strongly suggest that there is a clear advantage to the incorporation of low quantities of copper and chloride, improving the photosensitivity of the perovskite-based devices. However, this incorporation requires careful control and management as an excess of these ions may induce structural changes that in turn may tamper the optoelectronic properties. Considering the figure-of-merit of the devices, there is still room for improvement in the quantum efficiency of the devices, as our results yield below 0.1%. Thus, a comprehensive understanding and optimization of the chemical formulation are required to enhance the material quality of the perovskites. Also, the device fabrication technique can be significantly improved by using photolithography patterning steps to ensure quality contacts, as opposed to the colloidal painting used in this work.

## 4 Conclusions

The proposed low-cost and scalable synthesis approach yielded reproducible perovskite thin films with satisfactory photosensitivity, which renders them suitable for application in photodetectors. The three-step chemical solution methodology employed successfully enables the obtainment of all-inorganic perovskite films, with the resulting thin film having a chemical composition approximating CsPbI_1.5_Br_1.5_, which can be considered as a CsPb(Br_*x*_I_1–*x*_)_3_ perovskite solid solution with *x* = 0.5. Furthermore, the implementation of our proposed synthesis methodology facilitated the incorporation of copper and chloride ions, resulting in a perovskite thin film with an atomic composition of (Cs_0.95_:Cu_0.01_)PbI_1.5_Br_1.3_Cl_0.1_.

The results of this work suggest that the introduction of copper not only oxidizes the Cu^+^ ion during its incorporation into the perovskite structure but also induces structural modifications, like lattice compression and preferential growth via interstitial incorporation and Pb^2+^ substitution, significantly impacting the PL properties of the films. The remarkable enhancement in photodetector performance is evident, as demonstrated by a 5-fold increase in induced photocurrent and a substantial rise in responsivity in devices incorporating perovskite films with added copper and chloride ions.

This work not only contributes to the understanding of the structural and optoelectronic implications of copper and chloride incorporation in perovskite films but also highlights the potential for improving photodetector performance through the tailoring of the film composition. The industrially scalable nature of the proposed synthesis methodology highlights its applicability for large-scale production and, thus, the development of cost-effective and high-performance perovskite-based devices. Further research and optimization of the synthesis process, alongside an improved device fabrication methodology, will contribute to advancing the use of low-cost solution methods for future photodetector technologies.

## Supporting Information Available

FESEM micrograph of the chemically synthesized PbS_0.96_ film; XRD of PbS_0.96_ film; XPS spectrum for the S 2p and Pb 4f binding energy regions; PL spectrum of the precursor PbS thin films; FESEM micrograph of PbI_1.98_ film synthesized by a gas-solid reaction method; XRD of the PbI_1.98_ film; X-ray photoelectron spectra of PbI_1.98_ film; photoluminescence spectrum of PbI_1.98_ film; and determination of response time of the perovskite-based devices at a 13mW/cm^2^ light intensity using 1 s time pulses (PDF)
