# Supplementary material for: Photocurrent Enhancement by Copper Incorporation in Chemical-Solution-Synthesized Inorganic Lead Perovskite Thin Films
Source: ACS Omega. 2024 Mar 22;9(13):14985–96. doi: 10.1021/acsomega.3c09053 (PMC10993397; doi:10.1021/acsomega.3c09053)
Supplement: Supplementary file 1 — ao3c09053_si_001.pdf [file ao3c09053_si_001.pdf]

Supplementary file

Photocurrent enhancement by copper incorporation in chemical-solution-synthesized inorganic lead perovskite thin films

Igor Borges-Doren[a], Dagoberto Cabrera-German[a], Rodrigo Melendrez-Amavizca[b], Hailin Hu[c], Mérida Sotelo-Lerma[a,*]

[a] *Departamento de Investigación en Polímeros y Materiales, Universidad de Sonora, Hermosillo 83000, Mexico.*

[b] *Departamento de Investigación en Física, Universidad de Sonora, Hermosillo 83000, Mexico.*

[c] *Instituto de Energías Renovables, Universidad Nacional Autónoma de México, Temixco, Morelos, 62580, Mexico.*

*merida.sotelo@unison.mx

1 PbS synthesis characterization results:

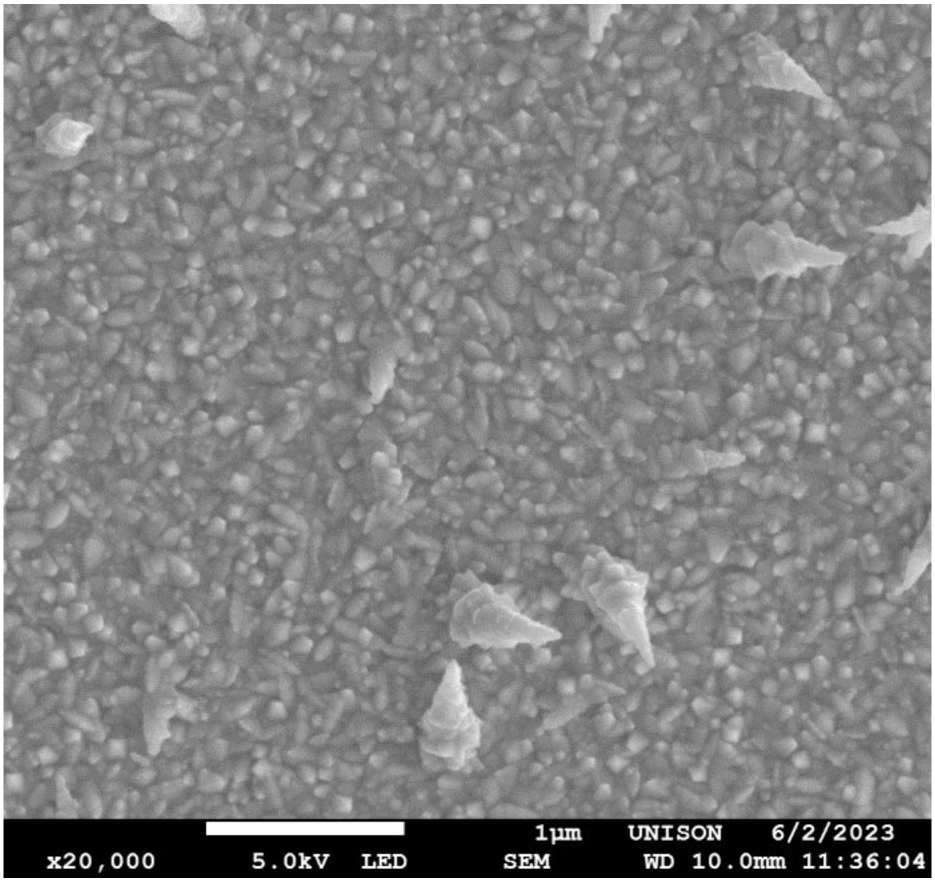

Figure S1. FESEM micrograph of the chemically synthesized $PbS_{0.96}$ film.

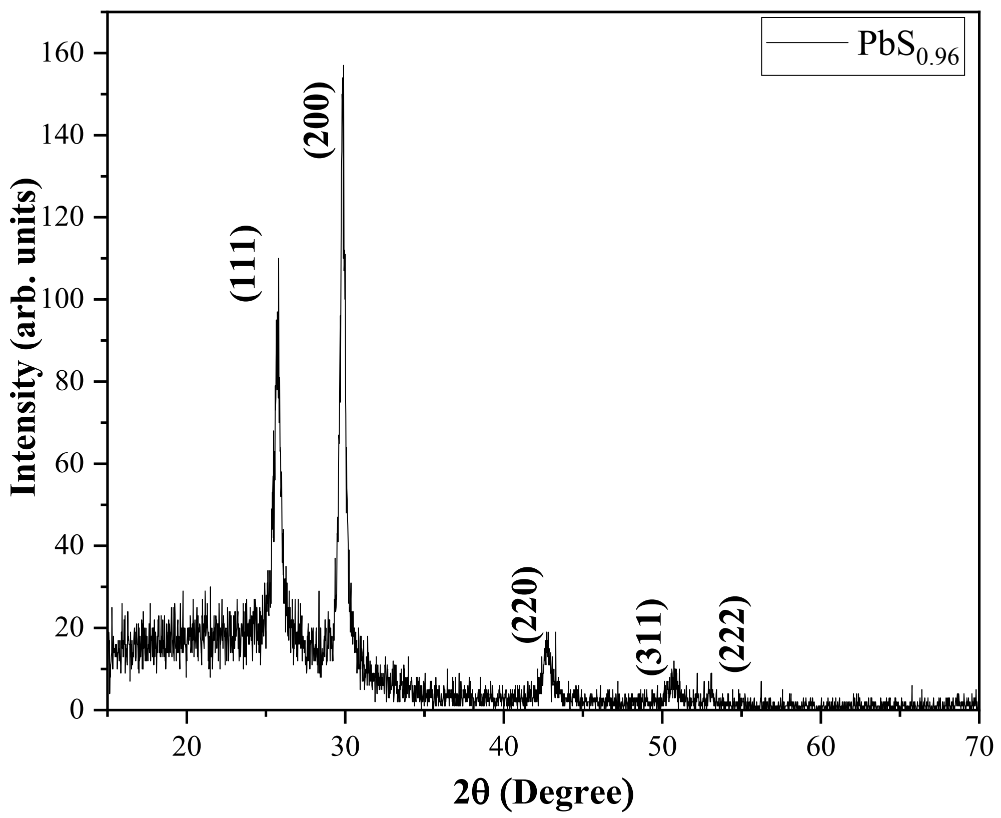

Figure S2. X-ray diffraction pattern obtained from the $PbS_{0.96}$ film.

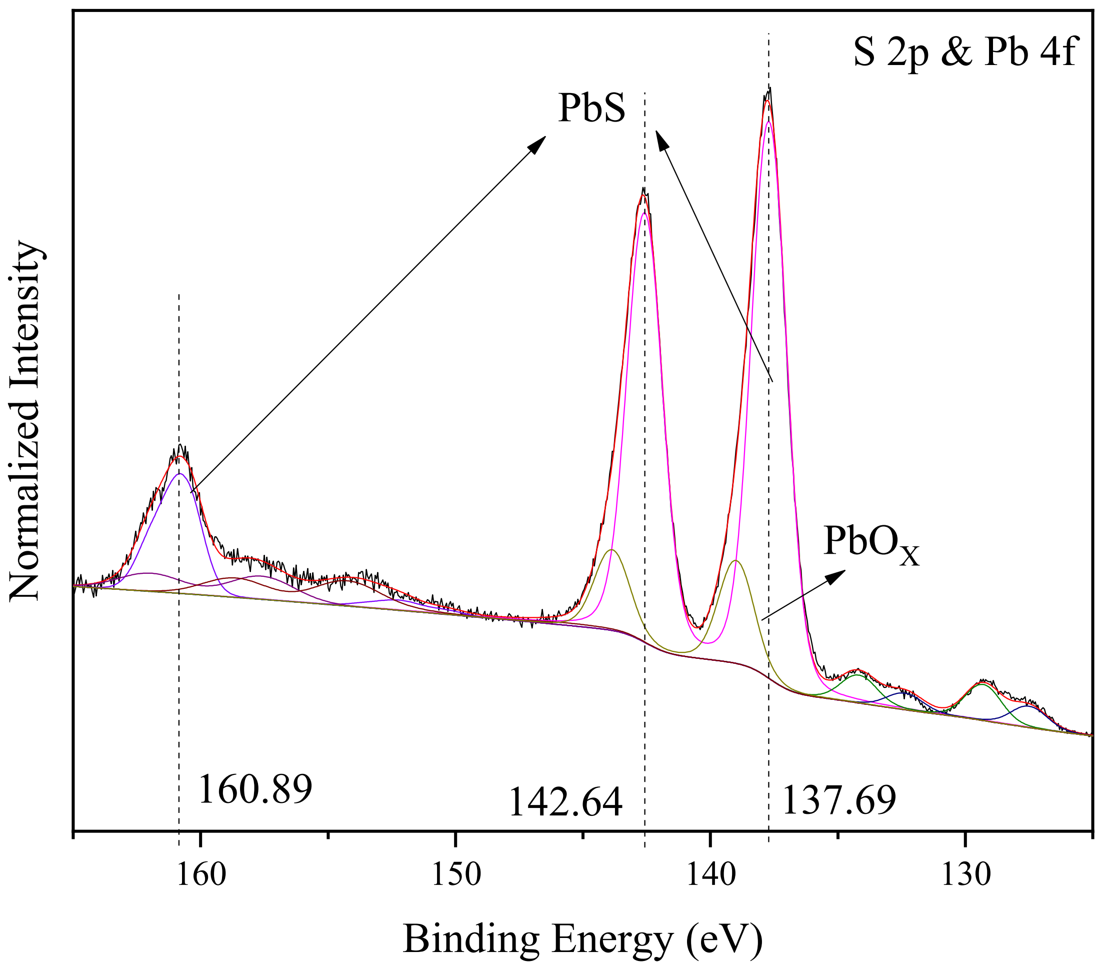

Figure S3. X-ray photoelectron spectra of the $PbS_{0.96}$ film.

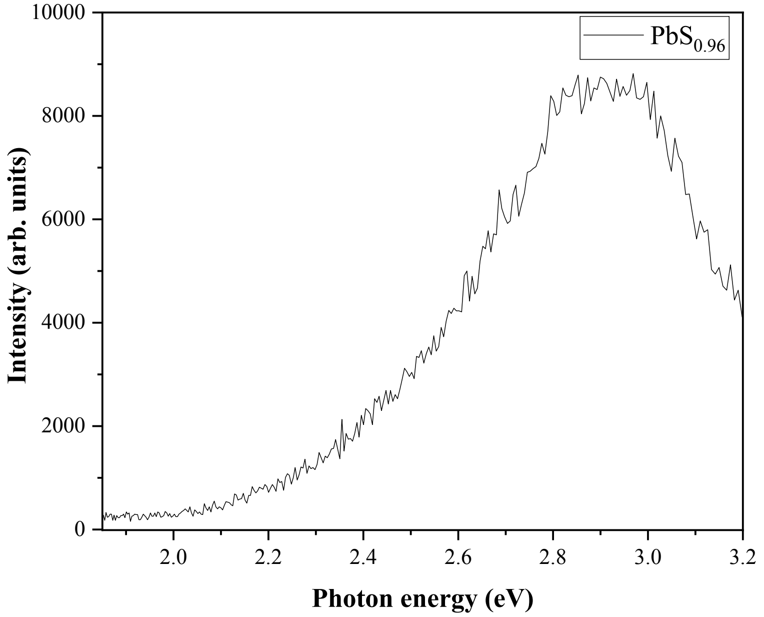

Figure S4. Photoluminescence spectrum of $PbS_{0.96}$ film.

2 PbI_2 synthesis characterization results

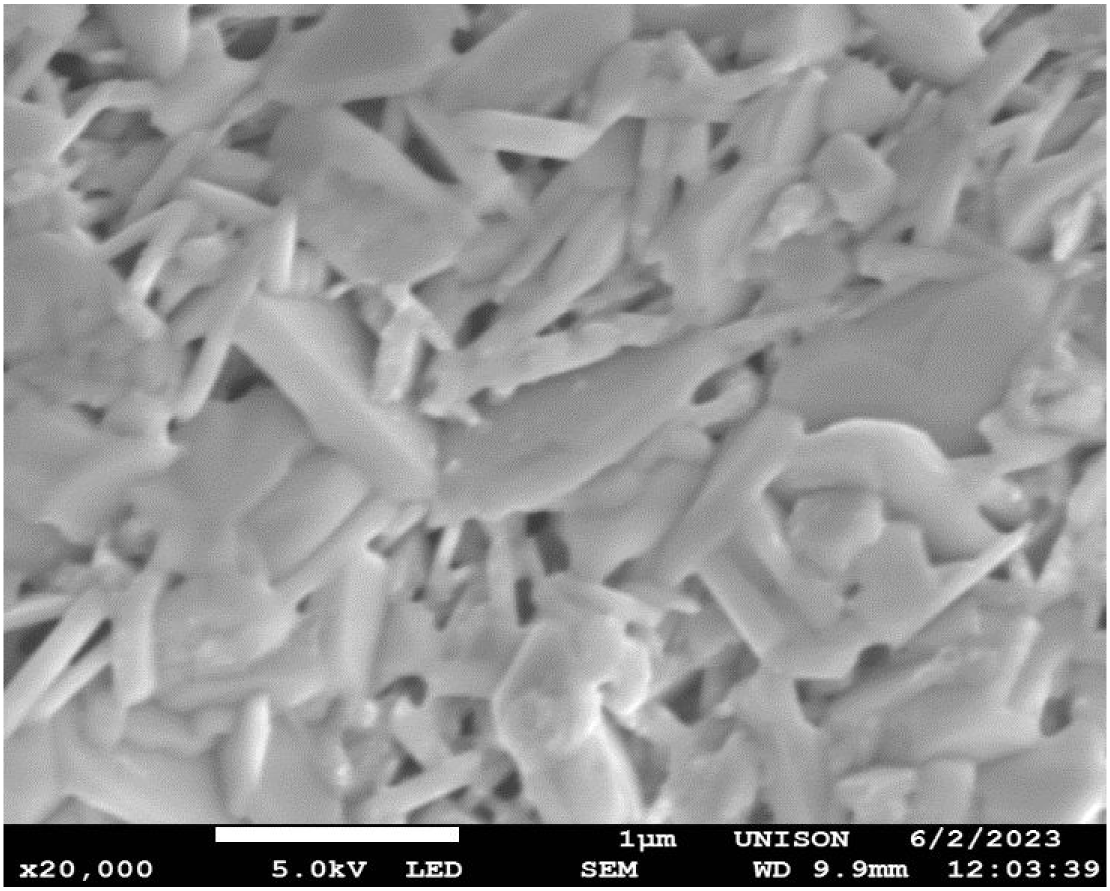

Figure S5. FESEM micrograph of $PbI_{1.98}$ film synthesized by a gas-solid reaction method.

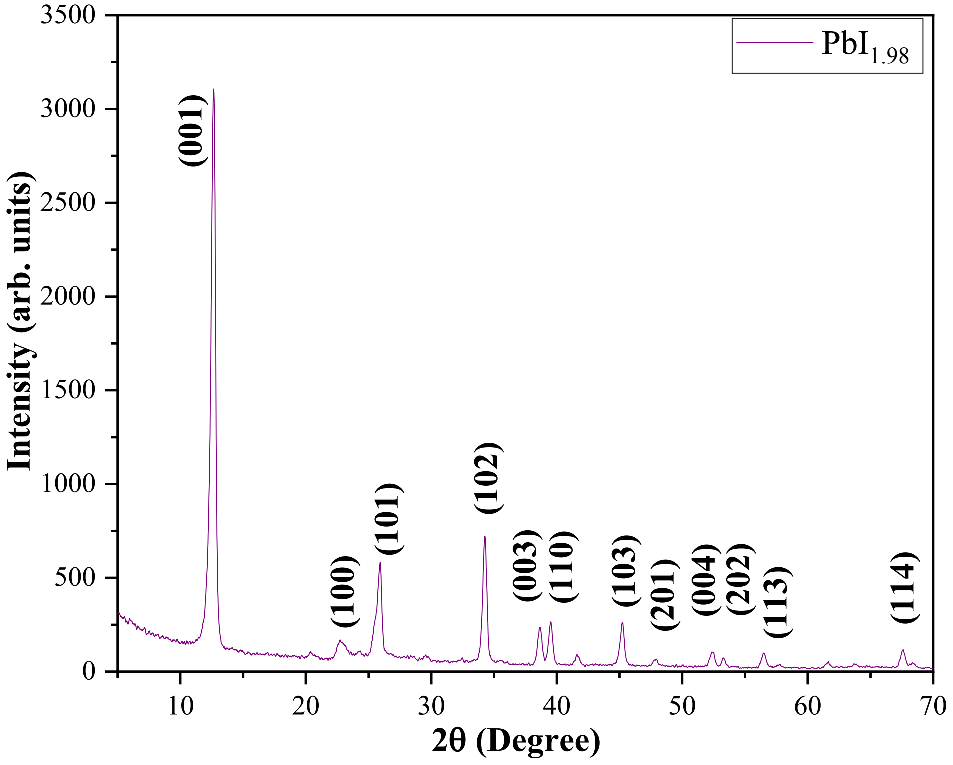

Figure S6. X-ray diffraction pattern of the $PbI_{1.98}$ film.

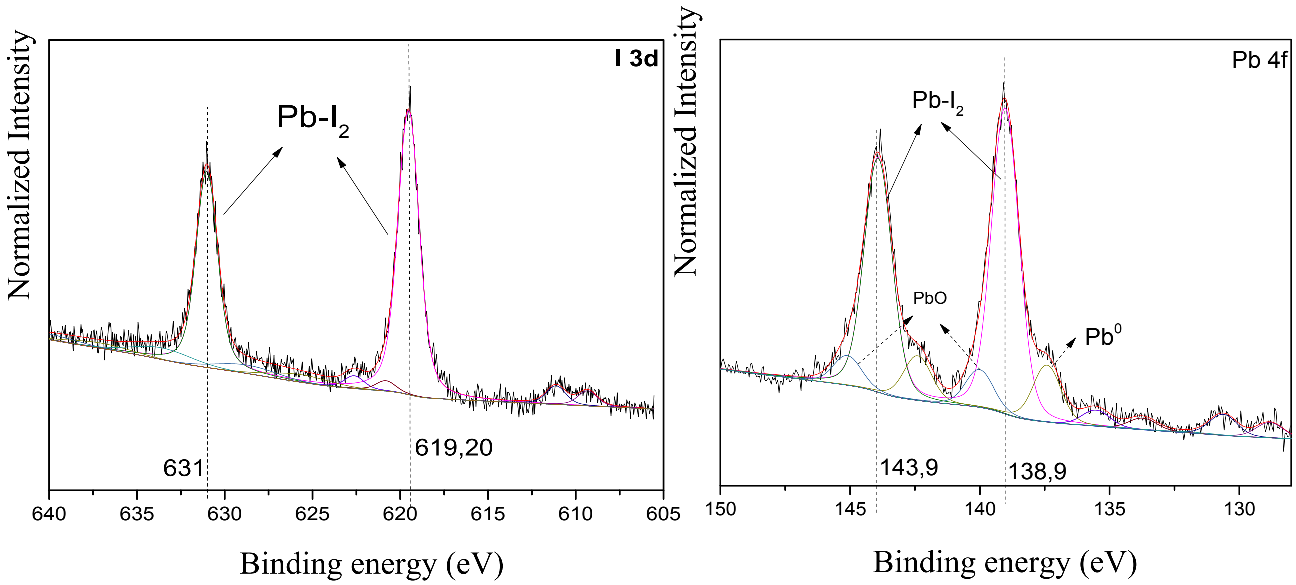

Figure S7. X-ray photoelectron spectra of PbI1.98 film

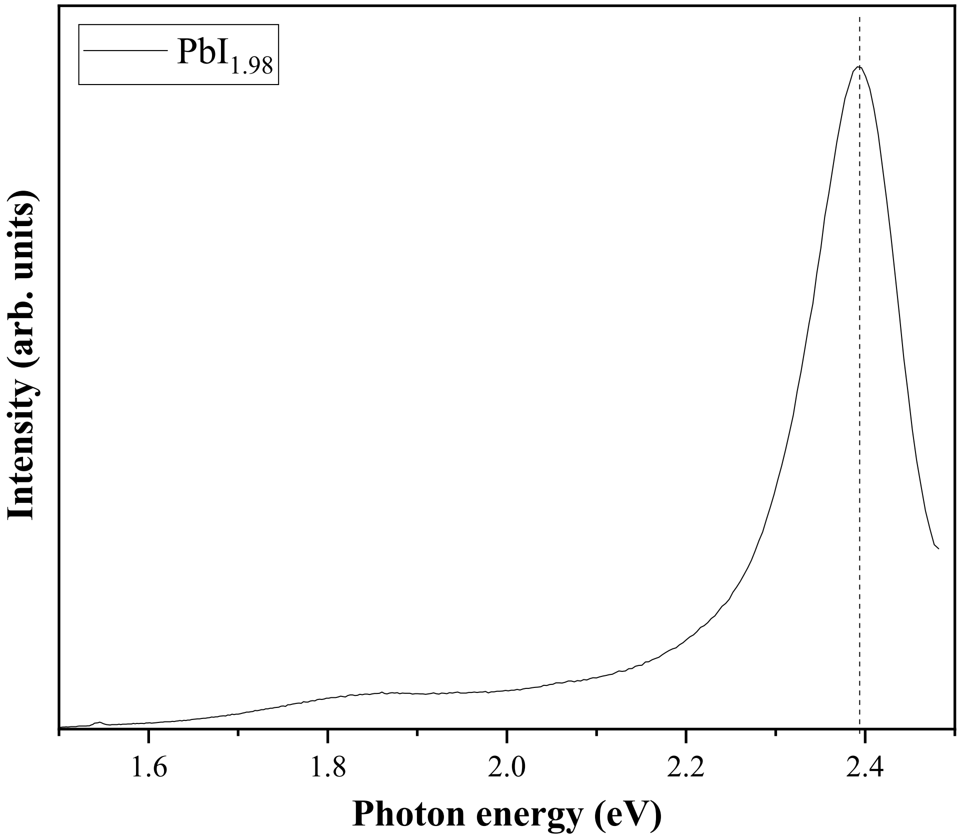

Figure S8. Photoluminescence spectrum of $PbI_{1.98}$ film.

3 Photodetector Figure of Merit

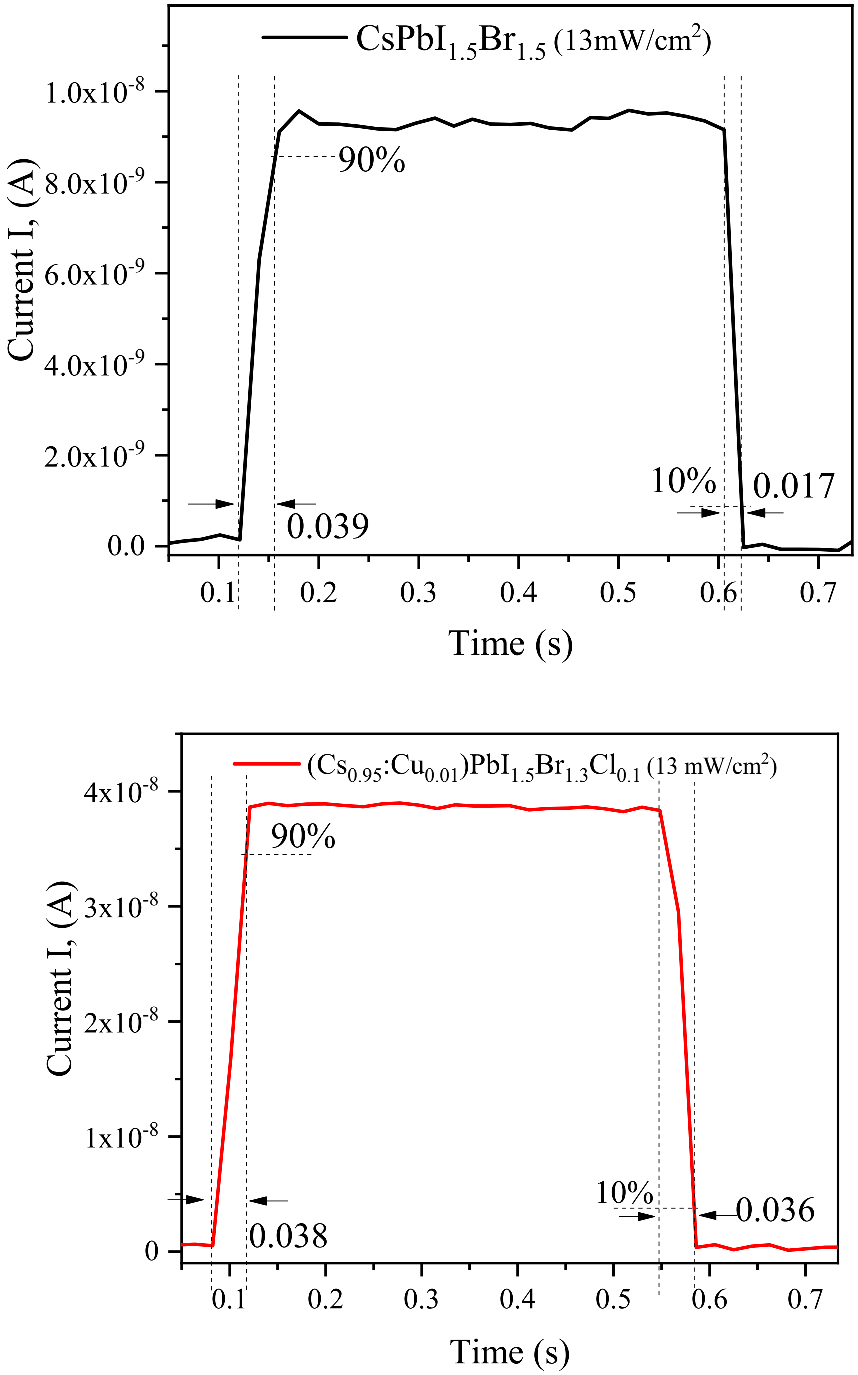

Figure S9. Determination of response time of the perovskite-based devices at a 13mW/cm^2 light intensity using 1 s time pulses.
